# Correction to “Physical Exercise Before Long‐Term Electrocardiogram May Enhance the Assessment of Nocturnal Heart Rate Variability in Young Individuals With Type 1 Diabetes Mellitus”

**DOI:** 10.1111/anec.70203

**Published:** 2026-05-27

Fridolfsson C, Blomstrand P, Engvall J, Thegerström J. Physical exercise before long‐term electrocardiogram may enhance the assessment of nocturnal heart rate variability in young individuals with type 1 diabetes mellitus. *Ann Noninvasive Electrocardiol*. 2025;30:e70125. https://doi.org/10.1111/anec.70125.

In the article title, the word “Melltius” was incorrectly spelled. This has been corrected to “Mellitus”.

We apologize for this error.

